# Circumferential myocardial strain in cardiomyopathy with and without left bundle branch block

**DOI:** 10.1186/1532-429X-12-2

**Published:** 2010-01-05

**Authors:** Yuchi Han, Jonathan Chan, Idith Haber, Dana C Peters, Peter J Zimetbaum, Warren J Manning, Susan B Yeon

**Affiliations:** 1 Departments of Medicine (Cardiovascular Division), Beth Israel Deaconess Medical Center, Harvard Medical School, Boston, MA, USA; 2 School of Medicine, University of Queensland, Brisbane, Australia; 3 Department of Medicine (Cardiology), University of Chicago Medical School, Chicago, IL, USA; 4 Department of Radiology, Beth Israel Deaconess Medical Center, Harvard Medical School, Boston, MA, USA

## Abstract

**Background:**

Cardiac resynchronization therapy (CRT) has been shown to decrease mortality in 60-70% of advanced heart failure patients with left bundle branch block (LBBB) and QRS duration > 120 ms. There have been intense efforts to find reproducible non-invasive parameters to predict CRT response. We hypothesized that different left ventricular contraction patterns may exist in LBBB patients with depressed systolic function and applied tagged cardiovascular magnetic resonance (CMR) to assess circumferential strain in this population.

**Methods:**

We determined myocardial circumferential strain at the basal, mid, and apical ventricular level in 35 subjects (10 with ischemic cardiomyopathy, 15 with non-ischemic cardiomyopathy, and 10 healthy controls). Patterns of circumferential strain were analyzed. Time to peak systolic circumferential strain in each of the 6 segments in all three ventricular slices and the standard deviation of time to peak strain in the basal and mid ventricular slices were determined.

**Results:**

Dyskinesis of the anterior septum and the inferior septum in at least two ventricular levels was seen in 50% (5 out of 10) of LBBB patients while 30% had isolated dyskinesis of the anteroseptum, and 20% had no dyskinesis in any segments, similar to all of the non-LBBB patients and healthy controls. Peak circumferential strain shortening was significantly reduced in all cardiomyopathy patients at the mid-ventricular level (LBBB 9 ± 6%, non-LBBB 10 ± 4% vs. healthy 19 ± 4%; both p < 0.0001 compared to healthy), but was similar among the LBBB and non-LBBB groups (p = 0.20). The LBBB group had significantly greater dyssynchrony compared to the non-LBBB group and healthy controls assessed by opposing wall delays and 12-segment standard deviation (LBBB 164 ± 30 ms vs. non-LBBB 70 ± 17 ms (p < 0.0001), non-LBBB vs. healthy 65 ± 17 ms (p = 0.47)).

**Conclusions:**

Septal dyskinesis exists in some patients with LBBB. Myocardial circumferential strain analysis enables detailed characterization of contraction patterns, strengths, and timing in cardiomyopathy patients with and without LBBB.

## Introduction

Cardiac resynchronization therapy (CRT) has been shown to improve symptoms, increase exercise capacity, decrease heart failure (HF) hospitalizations, and decrease mortality in patients with New York Heart Association (NYHA) Class III/IV HF with depressed systolic function, and a prolonged QRS in left bundle branch block (LBBB) morphology [1-3]. Recent data from patients with NYHA class I/II HF also demonstrated reduced HF hospitalization and reversal of left ventricular (LV) remodeling with CRT therapy [4]. However, 30-40% of patients who receive CRT therapy do not show significant clinical improvement [4-6]. As a result, there has been intense investigation to develop noninvasive parameters to predict CRT response [7-9]. While mechanical dyssynchrony assessed in the longitudinal axis of myocardial motion was shown to be predicative in single center trials [7-10], the multi-center PROSPECT trial failed to identify any echocardiographic dyssynchrony criteria to predict responders better than the clinical criteria [11].

Tagged cardiovascular magnetic resonance (CMR) is a noninvasive technique for measuring local deformation of the myocardium and quantitative assessment of mechanical dyssynchrony [12-14]. An advantage of tagged CMR circumferential strain (**ε**_cc_) measurements is the narrow and consistent normal range across different centers [15,16]. In addition, **ε**_cc _appears to be more sensitive to dyssynchrony than longitudinal strain in animal models [17]. **ε**_cc _patterns in healthy patients have been studied in detail [16,18]. We sought to examine **ε**_cc _patterns in patients with systolic dysfunction by applying tagged CMR.

## Methods

### Patient cohort

We studied twenty-five patients with systolic dysfunction referred for assessment of LV function and imaged between June 2006 and August 2009, including 10 patients with chronic ischemic cardiomyopathy (ICM) (age 64 ± 8 years, 90% male, LV ejection fraction (EF) 30 ± 6%) and 15 patients with non-ischemic dilated cardiomyopathy (non-ICM) (age 59 ± 11 years, 73% male, LVEF 27 ± 8%). All patients diagnosed with ICM had history of myocardial infarction and had coronary angiography demonstrating significant coronary artery disease involving at least two vessels. Eleven of 15 (73%) patients with non-ICM had coronary angiography demonstrating the absence of epicardial coronary artery stenoses. Four remaining patients were diagnosed as non-ICM with negative stress tests. Ten healthy adult subjects (age 38 ± 12 years, 50% male, EF 61 ± 4%) served as controls. The institutional Committee on Clinical Investigation approved the study protocol. Written informed consent was obtained from volunteers and was waived for existing clinical data sets.

### ECG analysis

All subjects had a standard 12-lead ECG performed within a median of 15 days (with interquartile range of [5.5, 25.5] days) of the CMR with no intervening change in clinical status. The QRS morphology was determined by an experienced electrophysiologist (PJZ) according to AHA/ACCF/HRS guidelines [19]. Briefly, LBBB was determined if the QRS duration was ≥120 ms, with presence of a broad monophasic R wave in I, or V5 and V6, absence of Q waves in leads I, V5, and V6, and the displacement of the ST segment and T waves in a direction opposite to the major deflection of the QRS complex. The QRS duration was determined by automated computerized measurements and confirmed manually.

### CMR

CMR studies were performed on a 1.5 T Philips Achieva MR scanner (Philips HealthCare, Best, NL), equipped with a 5-element cardiac coil. Breath-hold short-axis cine steady state free precession (SSFP) images covering the entire LV and long axis SSFP cine images covering the LV outflow tract were acquired as previously described [20].

Breath-hold ECG-gated tagged complementary spatial modulation of magnetization (CSPAMM) cine images at the basal, mid, and apical ventricular levels were obtained [21,22]. The mid ventricular level was prescribed at the mid-papillary muscle level. The center of the basal slice and the center of the apical slice were acquired 20 mm proximal and distal to the mid slice center, respectively. Scan parameters include spiral readout with 8 interleaves, 9 ms acquisition window, repetition time (TR)/echo time(TE)/flip angle(α) = 25 ms/3.6 ms/25°, field of view (FOV) = 320 mm × 320 mm, 10 mm slice thickness with 5 mm tag spacing, temporal resolution 25-35 ms, spatial resolution 2.5 × 2.5 × 10 mm.

Free-breathing, ECG-triggered phase contrast velocity sequences for aortic flow oriented in the axial plane at the level of the bifurcation of the pulmonary artery were acquired as previously described [23]. Sequence parameters were: TR/TE/α = 15 ms/6.5 ms/30°, FOV = 300 mm × 210 mm, matrix = 128 × 128, slice thickness 6 mm. Respiratory motion compensation was accomplished with the use of three signal averages.

2D breath-hold ECG-triggered late gadolinium enhancement (LGE) images were acquired in the same orientation as SSFP short axis images and long axis 2-chamber and 4-chamber orientations at 10-20 minutes post injection of 0.2 mmol/kg gadolinium-diethylenetriamine pentaacetic acid (Magnevist, Schering, Germany). Imaging parameters were: 2D spoiled gradient echo inversion recovery, TR/TE/α = 4.3 ms/1.5 ms/20°, FOV = 320 mm × 320 mm, matrix = 160 × 160, 8 mm slices with 2 mm gaps, partial echo, fat saturation, 1 RR between inversions, and two signal averages.

### Volumetric Analysis

Cardiac volumes were calculated in the standard fashion as previously described with papillary muscle included in the LV cavity volume [20]. Mitral regurgitation volume = LV stroke volume - aortic forward flow volume.

### Timing of systole

Systolic ejection begins when the aortic valve opens, as seen from the cine long axis LV outflow tract images and confirmed with phase contrast aortic flow curves. End of systole is defined as the time of aortic valve closure.

### CSPAMM image analysis

**Figure 1:**
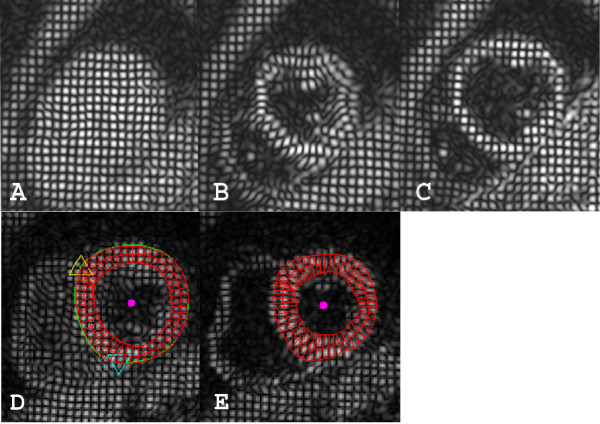
**CSPAMM images of mid ventricular short axis slice and analysis model**. A. Tags at the beginning of systole after ECG triggering. B. Same slice at end systole. C. Same slice at diastole with good persistence of tags. D and E show an example of CSPAMM images with the analysis model. D. In early systole, the epicardial border was manually drawn in green, endocardial border in red, superior right ventricular insertion site marked with a yellow triangle, and inferior insertion site marked with a blue triangle. The red donut is the analysis model with the myocardium partitioned in epi-myocardial, mid-myocardial, and endo-myocardial thirds shown in early systole in D and end-systole in E. Magenta dot is the center of the epicardial contour.

A customized software program (Cardiotool), written in MATLAB (MathWorks, Natick, MA), was used for semi-automated analysis of circumferential strain [24]. Endocardial and epicardial borders were drawn manually on the tagged images, and the right ventricular insertion sites were marked to indicate the outer borders of the anteroseptum and inferoseptum (Figure 1). The remaining myocardial slice was divided into anterior, anterolateral, inferolateral, and inferior segments according to the AHA 17-segment model. Circumferential strain from the mid-myocardial layer of each of the six segments of all three ventricular slices was analyzed.

### LGE image analysis

The presence of abnormal LGE in the myocardium was determined by the presence of high signal intensity (defined as 6 standard deviations higher than remote myocardium) in the short axis images and confirmed in the long axis images. The degree of LGE was determined by <50%, 50-75%, and >75% of LGE transmurality.

### Dyssynchrony assessment

Time to peak myocardial systolic strain in the mid-myocardial layer was identified for each segment by identifying the cardiac phase of peak systolic strain and multiplying by the temporal resolution in milliseconds. In myocardial segments with presystolic negative **ε**_cc _and systolic positive **ε**_cc_, the peak time was taken at the presystolic negative **ε**_cc_. For other segments, the peak time was taken at the maximal circumferential shortening, including the post-systolic shortening period. The absolute time difference between opposing walls (inferolateral wall to anteroseptum (IL-AS), anterior wall to inferior wall (A-I), and anterolateral wall to inferoseptum (AL-IS)) were obtained. The standard deviation of time to peak systolic strain in the basal and mid segments (T12SD) was determined.

### Statistics

Data were analyzed using the two-tailed Student's t-test to compare continuous variables and the Wilcoxon rank sum test to compare categorical variables. A two tailed p-value of < 0.05 was considered significant. In multiple group comparisons, Bonferroni correction was applied. All statistical analyses were performed with STATA Version 10 (STATcorp, TX, USA).

## Results

### Patient characteristics

Patient clinical characteristics are presented in Table 1. Twelve (48%) patients with ICM (n = 4) and non-ICM (n = 8) had LBBB with a mean QRS duration of 161 ± 10 ms. The thirteen non-LBBB patients had either normal QRS duration (< 100 ms) (n = 6) of 81 ± 5 ms or interventricular conduction delay (IVCD) (n = 7) with a mean QRS duration of 118 ± 14 ms. The healthy control subjects had normal QRS morphology and a mean QRS duration of 88 ± 12 ms.

**Table 1:** LBBB and non-LBBB patient characteristics:

	LBBB (n = 12)	Non-LBBB (n = 13)	p value
Age (years)	60 ± 10	62 ± 11	0.729

Sex (% male)	83%	77%	0.702

% ICM	33%	46%	0.532

LVEF (%)	27 ± 7	30 ± 7	0.327

LVEDV (ml)	307 ± 60	264 ± 60	0.086

LVESV (ml)	228 ± 56	189 ± 62	0.115

Mitral Regurgitation (ml)	7 ± 12	8 ± 8	0.817

QRS Duration (ms)	161 ± 10	106 ± 16*	< 0.0001

NYHA class (median)	2.5	1*	0.003

Hypertension	67%	85%	0.320

Diabetes	25%	31%	0.760

Hyperlipidemia	33%	69%	0.078

Tobacco Use	42%	46%	0.830

ACEI/ARB	100%	92%	0.337

Beta-blocker	100%	85%	0.165

Diuretic	67%	31%	0.078

Aspirin	67%	77%	0.589

Digoxin	42%	8%	0.059

Spironolactone	25%	15%	0.571

± standard deviation. ICM = ischemic cardiomyopathy. LVEF = left ventricular ejection fraction. LVEDV = left ventricular end-diastolic volume. LVESV = left ventricular end-systolic volume. NYHA = New York Heart Association Classification. ACEI/ARB = angiotensin converting enzyme inhibitor/angiotensin receptor blocker. *p < 0.05.

The LBBB and non-LBBB patient groups had similar age, gender, prevalence of hypertension, diabetes, hyperlipidemia, tobacco use, cardiac medication use, LVEF, LV volumes, and mitral regurgitation volume, but differed in their NYHA classification (Table 1).

### Myocardial scar

Among the four ICM patients with LBBB, three had inferior infarcts (two with LGE > 75%, one had LGE <50%) and one patient had both inferior and anterior infarcts (LGE = 50% of the septum, anterior and inferior walls). Among the six ICM patients with non-LBBB, four had anterior infarcts (3 subendocardial LGE, and one with > 75% of anteroseptum and anterior wall LGE) and two had inferior infarcts (one subendocardial LGE, one >75% LGE). No focal LGE was identified in patients with non-ICM.

### Circumferential strain patterns in LBBB

Three distinctive contractile patterns in LBBB patients were observed in the septum.

**Figure 2:**
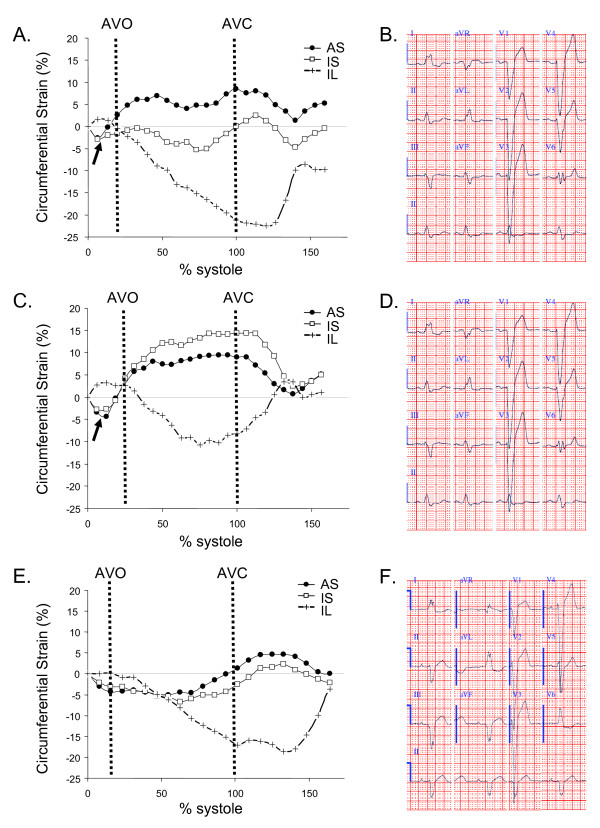
**Three different septal circumferential strain patterns in patients with LBBB**. AVO marks the opening of aortic valve and AVC marks the closure of aortic valve. A. Type Ia. Dyskinesis of the anteroseptum. Inferoseptum **ε**_cc _shortening is reduced. B. Corresponding ECG, QRS = 154 ms. C. Type Ib. Dyskinesis of the entire septum. D. Corresponding ECG, QRS = 150 ms. E. Type II. No dyskinesis but reduced septal **ε**_cc_. F. Corresponding ECG, QRS = 156 ms. Arrows point to presystolic contraction. AS = anteroseptum, IS = inferoseptum, and IL = inferolateral wall. AVO = aortic valve opening. AVC = aortic valve closure.

Type I: An initial negative **ε**_cc _in the septal segment before systolic ejection (as determined above) was present as presystolic contraction, followed by a positive **ε**_cc _reflecting stretching and dyskinesis of the septum. We further divided this type into two subtypes:

Type Ia. When Type I pattern is only present in the anteroseptum (Figure 2A).

Type Ib. When Type I septal pattern is present in both anteroseptum and inferoseptum (Figure 2C).

Type II. Decreased amplitude but no stretching in either of the septal segments (Figure 2E).

One LBBB patient had no basal or apical ventricular CSPAMM imaging. One patient was excluded from pattern analysis due to poor basal slice image quality. For the remaining 10 patients, 6 patients had fully concordant contractile pattern in the septum in all three slices. Two had basal and mid slice concordance and two had mid and apical slice concordance. Type II pattern was more prevalent in apical slices (50%) compared to 20% of mid slices and 10% of basal slices. Overall, the mid ventricular slice was the most representative of septal contraction pattern with 100% concordance with either the basal or apical slice or both. Among LBBB patients, 30% of had Type Ia, 50% of had Type Ib while 20% had Type II in the mid ventricular slice. These two Type II patients had QRS durations of 156 ms and 180 ms respectively. The different strain patterns cannot be predicted from ECG pattern or QRS duration in these LBBB patients (Figure 2B, D and 2F).

In addition, Type I pattern was present in the anterior segment in the mid slice in two of the three Type Ia patients and two of the five Type Ib patients. All remaining segments in all three slices had negative **ε**_cc _during systole.

### Circumferential strain pattern in patients without LBBB and healthy subjects

**Figure 3:**
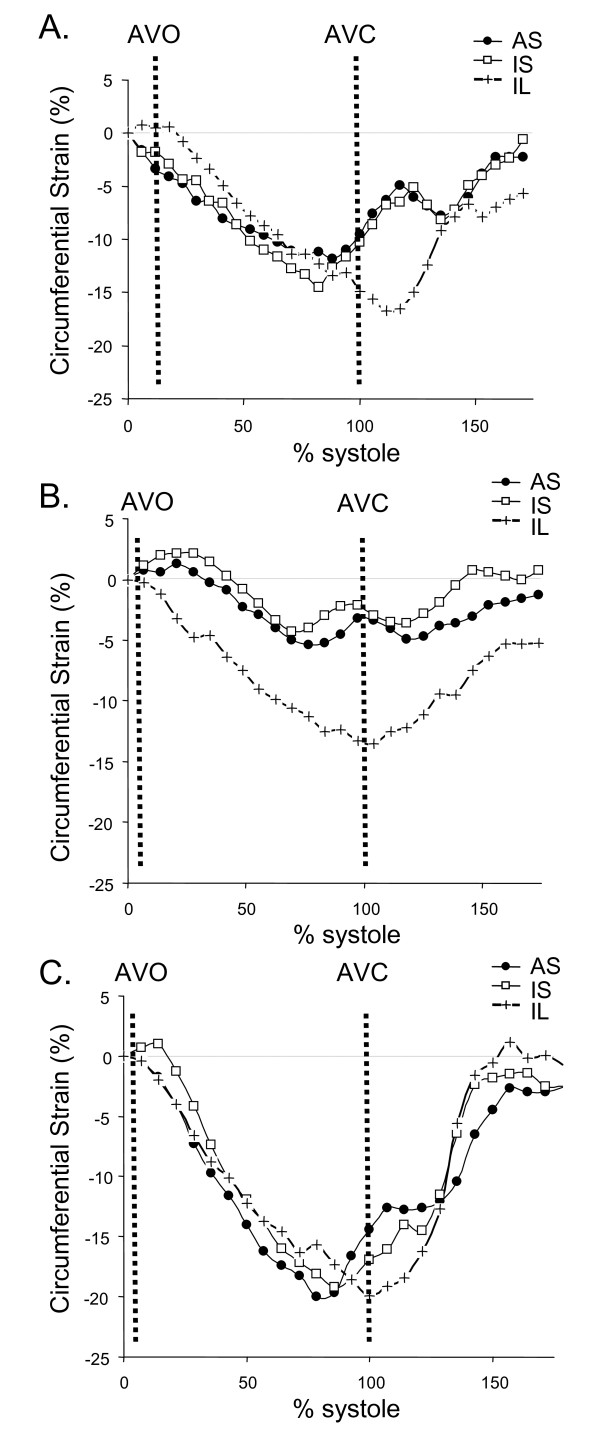
**Septal circumferential strain pattern in patients with non-LBBB (A, B) and healthy (C) subjects**. All subjects had normal contraction patterns with maximum negative circumferential strain reached in the septum earlier than in the inferolateral wall. A. Patient with interventricular conduction delay and QRS duration of 146 ms. B. Patient with a normal QRS duration of 98 ms. C. Healthy subject with a normal QRS duration of 98 ms. AS = anteroseptum, IS = inferoseptum, IL = inferolateral wall. AVO = aortic valve opening. AVC = aortic valve closure.

All patients without LBBB and all healthy subjects showed **ε**_cc _shortening in all segments throughout systolic contraction (Figure 3) in all slices at the basal, mid, and apical ventricular levels. The septal contractile pattern was similar in these subjects.

### Circumferential shortening patterns in ischemic vs. non-ischemic groups

**Figure 4:**
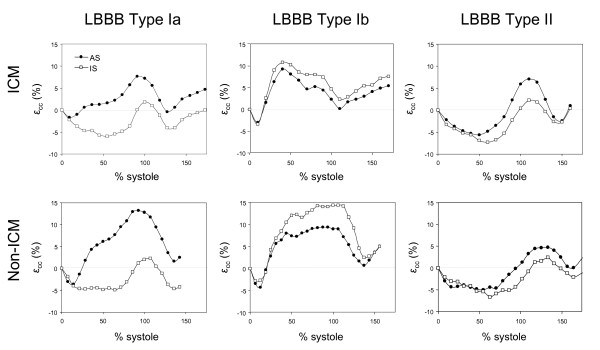
**All three types of septal contraction pattern can be observed in both ischemic (ICM) and non-ischemic (non-ICM) cardiomyopathy patients**. In LBBB Type Ia, only the anteroseptum is dyskinetic. In LBBB Type Ib, both anteroseptum and inferoseptum are dyskinetic. In LBBB Type II, neither anteroseptum nor inferoseptum is dyskinetic. AS = anteroseptum, IS = inferoseptum.

We found no difference in time to peak **ε**_cc _in ischemic and non-ischemic patients stratified by LBBB and non-LBBB. In three out of four patients with evidence for > 75% scar in the infarcted walls, there was no circumferential contraction (**ε**_cc _≈ 0). These three segments were excluded from timing analysis due to the absence of peak circumferential shortening. All remaining infarct segments were included in the analysis. One patient with ICM and non-LBBB had > 75% scar in the anteroseptum, but the **ε**_cc _in that segment was not near 0. In Figure 4, we show side by side the **ε**_cc _in the anteroseptum and inferoseptum of ICM and non-ICM patients with Type Ia, Type Ib, and Type II LBBB patterns.

### Circumferential shortening

**Figure 5:**
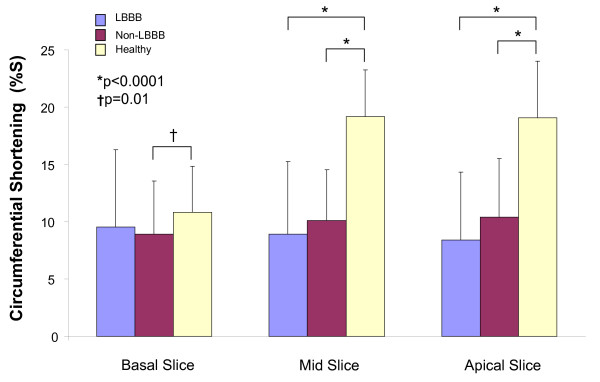
**Circumferential shortening magnitude in three ventricular slices**. Compared to healthy controls, both the LBBB and non-LBBB patients have overall significantly reduced **ε**_cc _%S in mid and apical ventricular slices. In the basal slice, non-LBBB patients had significantly reduced **ε**_cc _%S compared to healthy controls but not the LBBB patients. %S = % shortening.

A total of 24 of 618 segments (4%) from all subjects and all slices were excluded from analysis due to poor image quality. Cardiomyopathy patients showed reduced overall circumferential shortening (%S) in the mid slice (LBBB 9 ± 6%, non-LBBB 10 ± 4% vs. healthy 19 ± 4%) (both p < 0.0001 compared to healthy) and apical slice (LBBB 8 ± 6%, non-LBBB 10 ± 5% vs. healthy 19 ± 5%) (both p < 0.0001 compared to healthy) (Figure 5). Within the mid slice, in the non-LBBB group, %S was reduced in all segments compared to healthy controls (all p < 0.0001); while in the LBBB group, %S was reduced in all segments except the anterolateral wall as compared to controls. The LBBB patients had a significant reduction in %S of **ε**_cc _in the anteroseptum (3 ± 2% vs. 9 ± 4%) (p = 0.001) and anterior segment (6 ± 4% vs. 10 ± 2%) (p = 0.009) compared to non-LBBB patients, but not overall (p = 0.20) (Table 2).

**Table 2:** Comparison of circumferential myocardial strain percent shortening of all three ventricular slices in patients with and without LBBB, and healthy subjects.

		LBBB (%S)	Non-LBBB (%S)	Healthy (%S)	p(LBBB vs. non LBBB)*	p(LBBB vs. healthy)*	p(non-LBBB vs. healthy)*
Basal	AS	3 ± 2	6 ± 3	13 ± 4	0.005*	< 0.0001*	0.0009*
	
	A	9 ± 4	8 ± 4	11 ± 5	0.624	0.285	0.115
	
	AL	17 ± 3	15 ± 2	14 ± 3	0.121	0.066	0.502
	
	IL	16 ± 6	11 ± 4	11 ± 3	0.046	0.027	0.683
	
	I	9 ± 6	7 ± 3	8 ± 2	0.371	0.806	0.296
	
	IS	4 ± 2	6 ± 4	8 ± 3	0.177	0.003*	0.191

Mid	AS	3 ± 2	9 ± 4	18 ± 2	0.001*	< 0.0001*	< 0.0001*
	
	A	6 ± 4	10 ± 2	20 ± 3	0.009*	< 0.0001*	< 0.0001*
	
	AL	17 ± 4	14 ± 2	20 ± 3	0.071	0.047	= 0.0004*
	
	IL	16 ± 4	12 ± 4	24 ± 3	0.026	< 0.0001*	< 0.0001*
	
	I	7 ± 3	7 ± 5	16 ± 4	0.823	< 0.0001*	0.0003*
	
	IS	4 ± 3	7 ± 4	17 ± 3	0.036	< 0.0001*	< 0.0001*

Apical	AS	5 ± 4	9 ± 4	18 ± 3	0.012*	< 0.0001*	< 0.0001*
	
	A	6 ± 5	12 ± 5	18 ± 4	0.007*	< 0.0001*	0.004*
	
	AL	14 ± 4	14 ± 3	23 ± 5	0.891	0.0003*	0.0003*
	
	IL	16 ± 4	13 ± 4	22 ± 7	0.096	0.022	0.002*
	
	I	6 ± 3	7 ± 5	17 ± 3	0.565	< 0.0001*	< 0.0001*
	
	IS	5 ± 4	8 ± 5	16 ± 2	0.095	< 0.0001*	0.0001*

± standard deviation. *with Bonferroni correction for multiple comparisons, p < 0.017 was considered significant. AS = anteroseptum. A = anterior wall. AL = anterolateral wall. IL = inferolateral wall. I = inferior wall. IS = inferoseptum.

### Dyssynchrony

In patients with LBBB compared to non-LBBB or healthy subjects, there was significantly greater dyssynchrony of contraction as indicated by greater delays in the opposing walls IL-AS and AL-IS at all three ventricular levels, and T12SD (p ≤ 0.002) (Table 3). When comparing non-LBBB to healthy subjects, only basal IL-AS delay and mid AL-IS delay were significantly lengthened (p ≤ 0.002). The A-I time to peak strain had no significant delays in all three groups.

**Table 3:** Comparison of dyssynchrony measurements in three ventricular slices in patients with and without LBBB, and healthy subjects.

		LBBB	Non-LBBB	Healthy	p(LBBB vs. non LBBB)*	p(LBBB vs. healthy)*	p(non-LBBB vs. healthy)*
Basal	IL-AS (ms) (range)	391 ± 37 (325-425)	136 ± 79 (0-325)	45 ± 23 (0-75)	< 0.0001*	< 0.0001*	0.001*
	
	AL-IS (ms) (range)	284 ± 119 (150-425)	106 ± 54 (0-200)	68 ± 64 (0-175)	0.001*	0.0002*	0.171
	
	A-I (ms) (range)	122 ± 145 (0-400)	83 ± 85 (0-225)	68 ± 32 (0-100)	0.478	0.272	0.594

Mid	IL-AS (ms) (range)	353 ± 79 (245-425)	82 ± 44 (25-140)	69 ± 39 (0-125)	< 0.0001*	< 0.0001*	0.451
	
	AL-IS (ms) (range)	283 ± 108 (125-400)	114 ± 37 (25-175)	50 ± 46 (0-125)	0.0002*	< 0.0001*	0.002*
	
	A-I (ms) (range)	168 ± 164 (0-475)	69 ± 46 (0-175)	64 ± 55 (0-150)	0.097	0.084	0.791

Apical	IL-AS (ms) (range)	278 ± 118 (140-425)	61 ± 47 (0-325)	38 ± 41 (0-75)	< 0.0001*	< 0.0001*	0.260
	
	AL-IS (ms) (range)	224 ± 118 (150-425)	70 ± 63 (0-200)	54 ± 24 (0-175)	0.002*	0.001*	0.437
	
	A-I (ms) (range)	96 ± 110 (0-300)	48 ± 43 (0-150)	34 ± 20 (0-75)	0.200	0.093	0.601

T12SD (ms)		164 ± 30 (131-207)	70 ± 17 (51-103)	65 ± 17 (44-97)	< 0.0001*	< 0.0001*	0.467

Data presented as absolute time difference between the two walls ± standard deviation. *with Bonferroni correction for multiple comparisons, p < 0.017 was considered significant. IL-AS = inferolateral wall to anteroseptal wall. AL-IS = anterolateral wall to inferoseptal wall. A-I = Anterior wall to inferior wall.

### Interobserver and intraobserver variability

In six randomly selected subjects with 35 evaluable segments in the mid-ventricular slice, intraobserver variability and interobserver variability for peak systolic **ε**_cc _timing were 3 ± 7% and 7 ± 11%, respectively. The intraobserver variability and interobserver variability for systolic **ε**_cc _%S were 9 ± 8%, 9 ± 9%, respectively. Both observers on repeated analysis identified the same contractile patterns.

## Discussion

In our study of patients with LBBB pattern and depressed LV function, we found three types of septal myocardial circumferential strain patterns. Some patients with LBBB had severe mechanical dyssynchrony manifested as a specific contractile pattern with initial presystolic septal contraction during isovolumic contraction period followed by dyskinesis (positive **ε**_cc_) of the interventricular septum during the entire systole. This pattern was present in the anteroseptum (Type Ia) in 30% of patients, and in the entire septum in 50% of patients (Type Ib). The remaining 20% of LBBB patients had a normal contractile pattern, similar to non-LBBB cardiomyopathy patients and healthy controls, although the magnitude of contraction is significantly reduced in both groups of cardiomyopathy patients compared to healthy controls. All non-septal segments except four anterior segments in LBBB patients demonstrated **ε**_cc _shortening. Our finding of **ε**_cc _of 16-24% in normal healthy controls is consistent with literature findings in the mid-myocardial layer [15].

Detailed studies of healthy human subjects have demonstrated that the normal mechanical activation pattern starts at the septum and extends to the inferolateral wall [18]. We found presystolic mid-myocardial circumferential shortening (negative **ε**_cc_) during isovolumic contraction phase in part or the entire septum in patients with LBBB, followed by circumferential lengthening (positive **ε**_cc_) during systole. This altered pattern with early presystolic contraction and systolic dyskinesis contribute to increased severity of dyssynchrony. In our cohort of ICM and non-ICM patients, the presence or absence of LBBB had a dominating impact on the contractile pattern regardless of the etiology of the cardiomyopathy. In ICM patients, the only difference was the presence of akinesis in regions of transmural infarction in 3 segments. Our data for circumferential strain demonstrate that there are significant timing differences in opposing walls (IL-AS, AL-IS), and T12SD in patients with low EF and LBBB as compared to the patients with low EF but normal QRS or IVCD. The prognostic clinical significance of these dyssynchrony patterns to CRT remains to be determined.

In a 2D longitudinal strain by speckle-tracking study by Carasso et al, dyssynchrony timing was not the only predictor for CRT success [25]. In their cohort, the majority of non-responders to CRT had ≥1 segment of "holosystolic stretching" (dyskinesis) while all except one responder had no such segments [25]. In a non-responder, the holosystolic dyskinetic segment remained dyskinetic after CRT; in a responder, the early systolic dyskinetic segment had normal contractile pattern after CRT therapy [25]. Our study also demonstrates the existence of different pattern of contraction within LBBB, which may be as important as timing in the assessment of dyssynchrony in patients with systolic dysfunction. Further investigation is needed to determine whether the septal contractile patterns impact response to CRT.

This study has several limitations. We did not study patients with normal EF and LBBB, low EF and RBBB, or a low EF and narrow LBBB (120-149 ms). In a 2D speckle tracking study by Miyazaki et al, patients with LBBB and normal EF had increased opposing wall delays and increased standard deviation of time to peak longitudinal strain as compared to normal controls, but the increase was less than patients with reduced EF and normal QRS [26]. The patients with reduced EF < 35% and LBBB had the longest delays [26]. Our findings on circumferential strain in low EF and LBBB vs. non-LBBB patients are consistent with their results. Patients with different conduction abnormalities would be important to study in order to further define and understand the different mechanical contractile patterns associated with conduction abnormalities.

## Conclusions

We have demonstrated LBBB in some patients with systolic dysfunction is associated with dyskinesis of the anteroseptum or the entire septum, resulting in severe mechanical dyssynchrony. The strength of myocardial contraction is significantly reduced in cardiomyopathic patients regardless of conduction patterns. The recognition of the presence of different mechanical contraction patterns within the same conduction abnormality may be important for the selection of patients for CRT.

## List of abbreviations

CMR: cardiovascular magnetic resonance; CRT: Cardiac resynchronization therapy; HF: heart failure; LV: left ventricle; LBBB: left bundle branch block; NYHA: New York Heart Association; ICM: ischemic cardiomyopathy; SSFP: steady state free precession; TE: echo time; TR: repetition time; FOV: field of view; LGE: late gadolinium enhancement; CSPAMM: complementary spatial modulation of magnetization; IL-AS: inferolateral to anteroseptal; AL-IS: anterolateral to inferoseptal; A-I: anterior to inferior; IVCD: interventricular conduction delay; T12SD: Standard deviation of time to peak systole circumferential strain in the 12 segments of basal and mid ventricular slices; %S: percent shortening.

## Competing interests

The authors declare that they have no competing interests.

## Authors' contributions

YH designed the study, acquired data, analyzed and interpreted the data, and drafted the manuscript. JC analyzed and interpreted the data and critically revised the manuscript. IH and DCP participated in the analysis and interpretation of the data, and critical review of the manuscript. PJZ participated in the conception and design of study and ECG analysis. WJM participated in interpretation and critical review of the manuscript. SBY conceived the study, participated in the design, interpretation, and critical review of the manuscript. All authors read and approved the final manuscript.
